# Efficacy of ceftazidime-avibactam, meropenem-vaborbactam, and imipenem-relebactam combinations against carbapenemase-producing Enterobacterales in Switzerland

**DOI:** 10.1007/s10096-023-04647-0

**Published:** 2023-08-11

**Authors:** Patrice Nordmann, Maxime Bouvier, Laurent Poirel

**Affiliations:** 1 Medical and Molecular Microbiology, Faculty of Science and Medicine, University of Fribourg, Fribourg, Switzerland; 2 Swiss National Reference Center for Emerging Antibiotic Resistance, Fribourg, Switzerland

**Keywords:** Ceftazidime, Avibactam, Meropenem, Vaborbactam, Imipenem, Relebactam, Carbapenemase, Enterobacterales

## Abstract

Carbapenemase-producing in Enterobacterales (CPE) represent a critical health concern worldwide, including in Switzerland, leading to very limited therapeutic options. Therefore, our aim was to evaluate the susceptibility to the novel ß-lactam/ß-lactamase inhibitor combinations ceftazidime-avibactam, meropenem-vaborbactam, and imipenem-relebactam of CPE isolates recovered in Switzerland from 2018 to 2020. A total of 150 clinical CPE were studied including mainly *Klebsiella pneumoniae* (*n* = 61, 40.3%) and *Escherichia coli* (*n* = 53, 35.3%). The distribution of carbapenemases was as follows: KPC-like (32%), OXA-48-like (32%), NDM-like (24%), combinations of carbapenemases (10%), VIM-1 producers (*n* = 2), and a single IMI-1 producer. Overall, 77% of the strains were susceptible to meropenem-vaborbactam, 63% was susceptible to ceftazidime-avibactam, and 62% susceptible to imipenem-relebactam. Those data may contribute to optimize the choice of first line therapy for treating infections due to CPE.

Since 2000s, the worldwide spread of carbapenem-resistant Enterobacterales (CRE) has become a main public health concern recognized by many international bodies such as WHO (World Health Organization. Global priority list of antibiotic-resistant bacteria to guide research, discovery, and development of new antibiotics (Geneva: World Health Organization 2017). Evidence suggests that patients who are infected by carbapenem-resistant pathogens have an increased likelihood of mortality and morbidity as compared to those infected by carbapenem-susceptible pathogens [1]. This explains why it is important to identify CRE to optimize the treatment. Among CRE, carbapenemase-producing Enterobacterales (CPE) require early and accurate identification since corresponding genes are highly transferable at least among them and are co-resistant to many non-ß-lactam related antibiotics [2, 3]. Carbapenemases belong to one of three of the four groups of ß-lactamases, namely, Ambler classes A, B, and D. Class A includes mostly KPC enzymes, class B includes mostly NDM, VIM and IMP types, whereas class D carbapenemases in Enterobacterales include OXA-48-like (OXA-181, OXA-232, OXA-244…) [2].

Novel ß-lactam-ß-lactamase inhibitor (BL/BLI) combination therapies have been developed to mitigate the therapeutic difficulties for treatment of infections caused by carbapenemase producers [4]. They associate a cephalosporin or a carbapenem with a BLI, e.g., the combination of ceftazidime with the diazabicyclooctane avibactam, the combination of imipenem with the diazabicyclooctane relebactam, and the combination of meropenem with the boronic derivative vaborbactam. Avibactam inhibits the activity of KPC and OXA-48-like enzymes, whereas vaborbactam and relebactam inhibit the activity of Ambler class A carbapenemases only. None of the clinically available BL/BLI combinations inhibits the activity of class B carbapenemases. However, acquired resistance to those combos has been already often reported [5]. Taking into account the variability of resistance profiles to carbapenems exhibited by each carbapenemase producer and the differences in terms of inhibition profiles of each BLI, our goal was to evaluate the susceptibility to ceftazidime-avibactam, meropenem-vaborbactam, and imipenem-relebactam of a series of carbapenemase producers recovered in Switzerland from 2018 to 2020. Results of this study may contribute to optimize the choice of antibiotics once the carbapenemase producer is identified.

A total of 150 carbapenemase-producing Enterobacterales were included. They had been isolated from 2018 to 2020 in university hospitals, country hospitals, and private clinics and sent to our national reference center for emerging antibiotic resistance (NARA). They were representative of the carbapenemase producers circulating at the national level (Switzerland) considering that it is mandatory to send carbapenemase producers to this reference center since 2017. Strains belonged to several enterobacterial species such as *Escherichia coli*, *Klebsiella* spp., *Enterobacter* spp., *Hafnia alvei, Providencia* spp., and *Citrobacter* spp., being either recovered from infections or colonizations. Out of the 150 carbapenemase producers, there was a majority of *Klebsiella pneumoniae* (*n* = 61, 40.3%) and *E. coli* (*n* = 53, 35.3%). MICs of ceftazidime-avibactam, meropenem-vaborbactam, meropenem, and ceftazidime were determined by the E-test technique (bioMérieux, La Balme-Les-Grottes, France), and MICs of imipenem-relebactam and imipenem were determined by the broth microdilution technique (BMD) following the EUCAST guidelines and results interpreted according to the EUCAST guidelines (https://www.eucast.org/fileadmin/src/media/PDFs/EUCAST_files/Breakpoint_tables/v_11.0_Breakpoint_Tables.pdf). The reference strain *E. coli* ATCC 25922 was used as quality control for all testing. Carbapenemase production was determined using the Rapidec Carba NP test (bioMérieux). Strains positive with this test were screened by PCR (*bla*_KPC_, *bla*_OXA-48_, *bla*_NDM_, *bla*_VIM,_ *bla*_IMP,_ *bla*_IMI,_ *bla*_GES_). Sanger sequencing of amplified carbapenemase genes was performed by Microsynth AG (Microsynth AG, http://wwww.microsynth.com) to identify the exact carbapenemase gene alleles.

The distribution of carbapenemases was as follows: KPC (32%), OXA-48-like (32%), NDM (24%), combinations of dual carbapenemases (10%), plus two VIM-1 producers, and a single IMI-1 producer. The three main carbapenemase types (KPC, OXA-48, NDM) were all identified either in *K. pneumoniae* and in *E. coli*. Noteworthy, the carbapenemases OXA-244 and NDM-5 were extensively distributed among community *E. coli* isolates, as reported in many countries such as Germany or Switzerland [6–8]. Our strain collection also included three strains that produced different KPC variants conferring resistance to ceftazidime-avibactam, namely, KPC-41, KPC-46, and KPC-50 [9, 10] (Table 1).

**Table 1:** The distribution of carbapenemases

Species	Carbapenemase	Number	MIC μg/mL					
			MEM^*a*^	MEV^*b*^	CAZ^*c*^	CZA^*d*^	IMP^*e*^	I-R^*f*^
*Escherichia coli*	KPC-2	5	0.19 (1)	0.016 (3)	2 (2)	0.094 (1)	0.5 (1)	0.06 (1)
			0.5 (2)	0.023 (1)	4 (1)	0.125 (2)	2 (3)	0.125 (4)
			1 (1)	0.032 (1)	**6 (1)**	0.25 (2)	4 (1)	
			4 (1)		**48 (1)**			
	KPC-3	5	0.75 (2)	0.012 (1)	**8 (1)**	0.38 (2)	4 (3)	0.06 (2)
			4 (1)	0.016 (1)	**64 (1)**	0.5 (1)	**8 (2)**	0.125 (1)
			**32 (1)**	0.023 (3)	**192 (1)**	0.75 (1)		0.25 (2)
			**>32 (1)**		**>256 (2**)	1.5 (1)		
	NDM-5	15	1 (1)	1.5 (2)	**>256 (15)**	**>256 (15)**	2 (1)	2 (1)
			2 (1)	2 (1)			**8 (3)**	**4 (1)**
			3 (1)	4 (1)			**16 (8)**	**8 (2)**
			4 (1)	6 (1)			**32 (2)**	**16 (8)**
			**12 (1)**	**12 (3)**			**64 (1)**	**32 (3)**
			**24 (2)**	>**64 (7)**				
			**>32 (8)**					
	OXA-181	5	0.19 (2)	0.125 (1)	0.19 (1)	0.094 (1)	0.5 (1)	0.25 (3)
			0.5 (1)	0.19 (1)	3 (1)	0.125 (2)	1 (3)	0.5 (1)
			0.75 (1)	0.38 (1)	**6 (1)**	1 (1)	2 (1)	1 (1)
			1 (1)	0.75 (1)	**>256 (2)**	1.5 (1)		
				1 (1)				
	OXA-244	15	0.064 (3)	0.064 (1)	0.5 (1)	0.094 (1)	0.125 (2)	0.06 (1)
			0.094 (1)	0.094 (5)	1 (1)	0.125 (4)	0.25 (1)	0.125 (3)
			0.125 (3)	0.38 (5)	1.5 (2)	0.19 (6)	0.5 (7)	0.25 (6)
			0.25 (2)	0.5 (1)	2 (7)	0.25 (2)	1 (4)	0.5 (3)
			0.38 (3)	1 (3)	3 (2)	0.38 (1)	2 (1)	1 (2)
			0.5 (2)		**6 (1)**	0.5 (1)		
			1 (1)		**16 (1)**			
	OXA-48	6	0.19 (1)	0.19 (1)	0.064 (1)	0.023 (1)	0.5 (1)	0.25 (1)
			0.25 (1)	0.38 (3)	0.38 (1)	0.094 (1)	1 (2)	0.5 (1)
			0.38 (1)	0.75 (1)	0.5 (1)	0.125 (1)	2 (3)	1 (2)
			0.75 (1)	2 (1)	0.75 (1)	0.19 (1)		2 (2)
			1 (1)		1 (1)	0.25 (1)		
			2 (1)		2 (1)	0.38 (1)		
	OXA-181 + NDM-5	1	**>32 (1)**	**>64 (1)**	**>256 (1)**	**>256 (1)**	**8 (1)**	**8 (1)**
	OXA-48 + NDM-5	1	**>32 (1)**	**>64 (1)**	**>256 (1)**	**>256 (1)**	**16 (1)**	**16 (1)**
*Klebsiella pneumoniae*	KPC-2	10	**32 (1)**	0.023 (1)	**48 (2)**	0.5 (1)	**16 (1)**	0.06 (1)
			**>32 (9)**	0.25 (1)	**64 (2)**	0.75 (1)	**64 (5)**	0.125 (3)
				0.75 (1)	**128 (2)**	1 (4)	**128 (4)**	0.25 (3)
				1 (2)	**256 (1)**	1.5 (1)		0.5 (2)
				1.5 (1)	**>256 (3)**	2 (2)		1 (1)
				2 (2)		3 (1)		
				4 (1)				
				**>64 (1)**				
	KPC-3	12	3 (1)	0.012 (1)	**32 (1)**	0.75 (1)	4 (1)	0.06 (2)
			4 (1)	0.023 (2)	**192 (1)**	1 (3)	**8 (2)**	0.125 (2)
			**12 (1)**	0.032 (2)	**>256 (10)**	1.5 (2)	**16 (3)**	0.25 (4)
			**16 (1)**	0.047 (2)		2 (5)	**32 (1)**	0.5 (3)
			**32 (1)**	0.19 (1)		6 (1)	**64 (2)**	2 (1)
			**>32 (7)**	0.5 (2)			**128 (2)**	
				0.75 (1)			**256 (1)**	
				1 (1)				
	NDM-1	10	6 (4)	6 (1)	**>256 (10)**	**>256 (10)**	**8 (4)**	**8 (5)**
			8 (1)	8 (5)			**16 (3)**	**16 (2)**
			**32 (1)**	**16 (1)**			**32 (2)**	**32 (2)**
			**>32 (4)**	**>64 (3)**			**64 (1)**	**64 (1)**
	NDM-4	1	**24 (1)**	**32 (1)**	**>256 (1)**	**>256 (1)**	**16 (1)**	**32 (1)**
	NDM-5	1	2 (1)	4 (1)	**>256 (1)**	**>256 (1)**	**8 (1)**	**16 (1)**
	OXA-181	2	0.25 (1)	0.25 (1)	0.094 (1)	0.094 (2)	0.5 (1)	0.5 (1)
			1.5 (1)	1 (1)	0.19 (1)		2(1)	1 (1)
	OXA-232	2	8 (1)	8 (1)	**>256 (2)**	0.75 (1)	1 (1)	1 (1)
			**>32 (1)**	**>64 (1)**		2 (1)	**8 (1)**	**4 (1)**
	OXA-48	7	0.25 (1)	0.25 (1)	4 (1)	0.19 (1)	2 (3)	1 (1)
			1.5 (1)	1.5 (2)	**16 (2)**	0.25 (1)	**8 (2)**	2 (2)
			2 (1)	**12 (1)**	**64 (1)**	0.38 (2)	**128 (2)**	**8 (2)**
			**12 (1)**	**>64 (3**)	**192 (1)**	0.75 (1)		**64 (2**)
			**>32 (3)**		**>256 (2)**	1 (2)		
	VIM-1	1	**16 (1)**	2 (1)	**>256 (1)**	**>256 (1)**	**8 (1)**	**8 (1)**
	NDM-1 + OXA-232	3	**>32 (3)**	**>64 (3)**	**>256 (3)**	**>256 (3)**	**32 (3)**	**32 (2)** **64 (1)**
	NDM-1 + OXA-48	1	**>32 (1)**	**>64 (1)**	**>256 (1)**	**>256 (1)**	**>128 (1)**	**>128 (1)**
	NDM-5 + OXA-181	1	**>32 (1)**	**>64 (1)**	**>256 (1)**	**>256 (1)**	**64 (1)**	**64 (1)**
	OXA-181 + NDM-4	1	8 (1)	6 (1)	**>256 (1)**	**>256 (1)**	**16 (1)**	**16 (1)**
	OXA-181 + NDM-5	2	**>32 (2)**	**>64 (2)**	**>256 (2)**	**>256 (2)**	**128 (2)**	**64 (1)** **128 (1)**
	OXA-232 + NDM-5	1	**>32 (1)**	**>64 (1)**	**>256 (1)**	**>256 (1)**	**32 (1)**	**32 (1)**
	OXA-48 + NDM-1	2	**>32 (2)**	**>64 (2)**	**>256 (2)**	**>256 (2)**	**32 (2)**	**32 (2)**
	OXA-48 + NDM-5	1	8 (1)	8 (1)	**>256 (1)**	**>256 (1)**	**32 (1)**	**32 (1)**
*Klebsiella pneumoniae* CZA-resistant	KPC-41	1	1 (1)	0.06 (1)	**>128 (1)**	**>128 (1)**	2 (1)	0.25 (1)
	KPC-46	1	2 (1)	0.5 (1)	**>128 (1)**	**128 (1)**	0.5 (1)	0.5 (1)
	KPC-50	1	4 (1)	0.12 (1)	**>128 (1)**	**128 (1)**	**16 (1)**	0.5 (1)
*Klebsiella oxytoca*	KPC-2	1	**32 (1)**	0.023 (1)	**24 (1)**	1 (1)	**64 (1)**	0.25 (1)
	KPC-3	4	2 (1)	0.023 (3)	**48 (1)**	0.75 (1)	1 (1)	0.064 (1)
			8 (1)	0.032 (1)	**128 (1)**	1.5 (2)	**8 (3)**	0.125 (1)
			**12 (1)**		**>256 (2)**	2 (1)		0.5 (2)
			**16 (1)**					
	OXA-48	2	0.25 (1)	0.25 (1)	0.125 (1)	0.094 (1)	2 (1)	0.5 (1)
			4 (1)	2 (1)	0.5 (1)	0.125 (1)	4 (1)	2 (1)
*Klebsiella aerogenes*	KPC-3	3	4 (1)	0.023 (2)	**24 (1)**	0.5 (1)	**16 (3)**	0.064 (1)
			**32 (1)**	0.047 (1)	**48 (1)**	0.75 (1)		2 (2)
			**>32 (1)**		**96 (1)**	1 (1)		
*Enterobacter cloacae*	KPC-2	1	**24 (1)**	0.032 (1)	**32 (1)**	0.75 (1)	**8 (1)**	0.25 (1)
	KPC-3	2	4 (1)	0.032 (1)	**>256 (2)**	1.5 (1)	**16 (2)**	0.25 (2)
			6 (1)	0.047 (1)		2 (1)		
	NDM-1	6	1 (2)	0.5 (1)	**>256 (6)**	**>256 (6)**	2 (2)	2 (2)
			2 (1)	0.75 (1)			4 (2)	**4 (2)**
			3 (2)	1 (2)			**8 (1)**	**8 (1)**
			**12 (1)**	4 (1)			**32 (1)**	**64 (1)**
				8 (1)				
	NDM-5	1	6 (1)	6 (1)	**>256 (1)**	**>256 (1)**	**16 (1)**	**16 (1)**
	NDM-7	1	**>32 (1)**	**>64 (1)**	**>256 (1)**	**>256 (1)**	**16 (1)**	**32 (1)**
	OXA-204	1	0.19 (1)	0.094 (1)	0.25 (1)	0.125 (1)	0.25 (1)	0.125 (1)
	OXA-48	4	0.5 (1)	0.38 (1)	0.5 (1)	0.25 (2)	1 (2)	1 (3)
			0.75 (1)	0.75 (2)	**16 (1)**	0.38 (1)	2 (2)	2 (1)
			1.5 (1)	1 (1)	**32 (1)**	0.5 (1)		
			2 (1)		**64 (1)**			
	IMI-1	1	2 (1)	0.016 (1)	0.25 (1)	0.125 (1)	**256 (1)**	**32 (1)**
	VIM-1	1	**>32 (1)**	**>64 (1)**	**>256 (1)**	**>256 (1)**	**8 (1)**	**8 (1)**
	OXA-48 + NDM-1	1	**12 (1)**	8 (1)	**>256 (1)**	**>256 (1)**	**32 (1)**	**32 (1)**
*Providencia stuartii*	NDM-1	1	1 (1)	0.38 (1)	**>256 (1)**	**>256 (1)**	**32 (1)**	**32 (1)**
*Citrobacter freundii*	KPC-2	3	6 (1)	0.094 (2)	**12 (2)**	0.75 (1)	**8 (1)**	0.5 (2)
			8 (1)	0.125 (1)	**32 (1)**	0.5 (1)	**32 (1)**	1 (1)
			**>32 (1)**			1.5 (1)	**64 (1)**	
	OXA-181	1	**12 (1)**	4 (1)	**>256 (1)**	1 (1)	2 (1)	2 (1)
*Citrobacter amalonaticus*	OXA-181	1	0.125 (1)	0.094 (1)	**6 (1)**	0.094 (1)	0.125 (1)	0.125 (1)
*Hafnia alvei*	OXA-48	1	3 (1)	4 (1)	1.5 (1)	0.38 (1)	**8 (1)**	**8 (1)**

^a^MEM: Meropenem. Breakpoint: *S* ≤ 2, *R* > 8

^b^MEV: Meropenem + vaborbactam at fixed concentration 8 μg/mL. Breakpoint: *S* ≤ 8

^c^CAZ: Ceftazidime. Breakpoint: *S* =<1, *R* > 4

^d^CZA: Ceftazidime + avibactam at fixed concentration 4 μg/mL. Breakpoint: *S* ≤ 8

^e^IMP: Imipenem. Breakpoint: *S* ≤ 2, *R* > 4

^f^I-R: Imipenem + relebactam at fixed concentration 4 μg/mL. Breakpoint: *S* ≤ 2

^a,b,c,d,e,f^(*n*) number of strains

^a,b,c,d,e,f^Bold means resistance categorization

Overall, 77% of the strains were susceptible to meropenem-vaborbactam, as compared to only 37% of susceptibility to meropenem alone. By comparison, only 10% and 33% of the isolates were susceptible to ceftazidime and imipenem, respectively, and only 63.3% and 62% of the isolates were susceptible to ceftazidime-avibactam and imipenem-relebactam, respectively. In *E. coli*, all strains that produced an OXA-48 like enzyme (without combination with any other carbapenemase) were susceptible to ceftazidime-avibactam, meropenem-vaborbactam, and imipenem-relebactam. For those strains, susceptibilities to meropenem-vaborbactam and imipenem-relebactam were basically the consequence of susceptibility to meropenem or imipenem (thus regardless the inhibitory effect of vaborbactam, or relebactam), whereas susceptibility to ceftazidime-avibactam was mainly related to the inhibitory activity of avibactam against OXA-48-like enzymes. Interestingly, several NDM-producing *E. coli* or *K. pneumoniae* remaining susceptible to meropenem-vaborbactam and/or imipenem-relebactam were resistant to ceftazidime-avibactam. Such discrepancy could actually be explained by their susceptibilities to meropenem and/or imipenem. Such observation is noteworthy since the prevalence rate of NDM producers in *Enterobacterales* is increasing in Switzerland as well as many other European countries [11]. As expected, KPC producers remained susceptible to ceftazidime-avibactam, meropenem-vaborbactam, and imipenem-relebactam, with the exception of the three *K. pneumoniae* producing KPC variants known to confer resistance to ceftazidime-avibactam, as mentioned above (Table 1). Susceptibility to meropenem, imipenem, meropenem-vaborbactam, and imipenem-relebactam of those latter ceftazidime-avibactam-resistant KPC producers correspond to a now commonly observed phenotype, with resistance to ceftazidime-avibactam being paradoxically associated with an increased susceptibility to carbapenems when related to production of some peculiar KPC variants [12].

Conversely, five out of eleven OXA-48-like producing *K. pneumoniae* showed resistance to meropenem and meropenem-vaborbactam, and to imipenem and imipenem-relebactam, whereas they all remained susceptible to ceftazidime-avibactam. This result was likely related to the well-recognized good inhibitory activity of avibactam toward Ambler class A ß-lactamases being responsible for resistance to ceftazidime (owing that OXA-48-like enzyme do not compromise the efficacy of that cephalosporin), and the commonly observed significant impact of outer membrane permeability defects on carbapenem resistance, as previously reported [13].

Among NDM-producing *E. cloacae*, meropenem-vaborbactam and imipenem-relebactam were more efficient than ceftazidime-avibactam, in line with the low MIC values of meropenem and imipenem but the high MIC values of ceftazidime of those strains.

As expected, for isolates producing a combination of OXA-48-like and NDM enzymes, resistance to ceftazidime-avibactam, meropenem-vaborbactam, and imipenem-relebactam was observed, with the exception of 3 out of 15 strains. These exceptions actually corresponded to isolates remaining susceptible to meropenem and consequently also to meropenem-vaborbactam.

A total of 21 strains were resistant to ceftazidime-avibactam and imipenem-relebactam but remained susceptible to meropenem-vaborbactam, namely, 18 producers of class B ß-lactamases (17 NDM, 1 VIM), and 3 dual carbapenemase producers (OXA-48 + NDM). Noteworthy, the hydrolytic activities of MBLs are not affected by all those ß-lactamase inhibitors. Therefore, respective MICs correlate with the susceptibility to meropenem and imipenem. Some paradoxical differences in categorizations for meropenem-vaborbactam and imipenem-relebactam can be explained by the difference in the respective breakpoints, being 8 mg/L for meropenem-vaborbactam and 2 mg/L for and imipenem-relebactam.

A total of 23 isolates remained susceptible to meropenem-vaborbactam but resistant to imipenem-relebactam, namely, (i) a single isolate producing the class A carbapenemase IMI-1, (ii) a single isolate producing the class D carbapenemase OXA-48 but showing MICs of imipenem and imipenem-relebactam at 8 mg/L (close to the breakpoints), and 21 isolates producing class B carbapenemases (including three isolates co-producing an OXA-48-like class B carbapenemase) among which 12 isolates also exhibited MICs of meropenem-vaborbactam and imipenem-relebactam being at only one dilution from the breakpoint, being respectively at 2 mg/L for imipenem-relebactam and 8 mg/L for meropenem-vaborbactam according to the EUCAST.

Interestingly, a single KPC-2-producing *K. pneumoniae* isolate showed resistance to meropenem-vaborbactam but remained susceptible to imipenem-relebactam and to ceftazidime-avibactam.

As a conclusion, meropenem-vaborbactam was significantly more effective than ceftazidime-avibactam and imipenem-relebactam against carbapenemase-producing Enterobacterales recently recovered in Switzerland. Despite the fact that vaborbactam and relebactam do not inhibit the hydrolytic activity of OXA-48, meropenem-vaborbactam and imipenem-relebactam showed a significant efficacy (even though lower than ceftazidime-avibactam) against OXA-48 producers. This is mainly related to the low MIC values of meropenem and imipenem of those OXA-48 producers, being consistent with the relatively low carbapenemase activity of OXA-48-type ß-lactamases [12]. Also, meropenem-vaborbactam was more effective than ceftazidime-avibactam against NDM producers, being the consequence of the susceptibility to meropenem observed for those isolates, and basically not to the inhibitory action of vaborbactam. corresponding to isolates remaining susceptible to meropenem basically. Even if not evaluated in the present study, since still not available as a therapeutical option (unless ceftazidime-avibactam plus aztreonam would be delivered), it is obviously of great significance to consider aztreonam-avibactam as an interesting alternative against those NDM-producing isolates.

This study provides susceptibility data to the recently-launched ß-lactam/ß-lactamase inhibitor combinations that may contribute to optimize the choice of first line therapy for treating infections due to carbapenemase producers. Hence, among the most relevant observations generated by this study, ceftazidime-avibactam might not always be preferred over meropenem-vaborbactam and imipenem-relebactam as a treatment option for treating infections due to KPC producers, as previously considered. This means that not only accurate MIC susceptibility data must be evaluated but also that further work is required to evaluate (i) the frequency of occurrence of resistant mutants upon treatment and (ii) the mechanisms by which such acquired resistance might be achieved. Indeed, such additional knowledge (therefore including local epidemiology data) would be crucial to determine whether either meropenem-vaborbactam, imipenem-relebactam, or ceftazidime-avibactam resistance shall be considered when choosing the optimal treatment for treating CRE infections [13, 14].

## Data Availability

All data generated through this study can be available upon request.

## Declarations

### Disclaimer

The funder was not involved in the study design, collection, analysis, interpretation of data, the writing of this article, or the decision to submit it for publication.
